# Evaluating attention deficit and hyperactivity disorder (ADHD): a review of current methods and issues

**DOI:** 10.3389/fpsyg.2025.1466088

**Published:** 2025-02-24

**Authors:** Hande Musullulu

**Affiliations:** Faculty of Health Sciences, Universidad del Atlántico Medio, Las Palmas de Gran Canaria, Spain

**Keywords:** ADHD, diagnosis, evaluation tools, assessment, rating scales

## Abstract

Attention-deficit/hyperactivity disorder (ADHD) is a common and complex neurodevelopmental disorder that affects individuals across the lifespan. This review provides an overview of the DSM diagnostic criteria for ADHD and discusses recommended considerations for the diagnosis of ADHD in children, adolescents, and adults. Its complexity requires careful consideration in the diagnostic process due to heterogeneity in clinical presentation and symptom overlap with other conditions. Commonly used assessment tools, including clinical interviews, rating scales and continuous performance tasks are reviewed with a focus on their psychometric qualities. Limitations of current diagnostic techniques, including issues related to gender bias, comorbidities and the importance of differential diagnosis are also reviewed. Improvements in the consistency and accuracy of ADHD diagnosis may be achieved by addressing these factors and evaluating the practical application of diagnostic tools.

## 1 Background and prevalence of ADHD

Attention deficit and hyperactivity disorder (ADHD) is defined as a neurodevelopmental condition by American Psychiatric Association (2013), the Diagnostic and Statistical Manual of Mental Disorders V (DSM-5), and the World Health Organization International Classification of Diseases 11 (ICD-11) (World Health Organization, 2019). It is characterized by impaired attention, hyperactivity, and impulsivity, resulting in negative outcomes in multiple settings of one’s life. Due to its clinical presentation and vulnerability to comorbid diseases, ADHD is associated with increased risk of substance use, health issues, accidents and behavioral addictions among others (Pozzi et al., 2018; Rosenbloom and Wultz, 2011).

It is one of the most common neurodevelopmental disorders which affects 5.9% of children and adolescents (Faraone et al., 2021) and 3.10% among adults with inattentive presentation as the most common type (Ayano et al., 2023). Studies on ADHD prevalence usually focus on children and adolescents and the data on the adult ADHD is quite limited. This is because ADHD symptoms are more clinically apparent and easily identified in children and adolescents than adults. However, with the recent developments and approaches, studies on adult ADHD data are also on the rise. For instance, a meta-analysis estimated ADHD prevalence among adult based on those who reported ADHD symptoms since childhood (persistent ADHD) and those who reported ADHD symptoms in adulthood, regardless of the childhood onset. The results revealed that the worldwide prevalence for persistent adult ADHD was 2.58% and symptomatic adult ADHD was reported to be 6.76% (Song et al., 2021). While persistent adult ADHD reflects accurate epidemiological data aligned with the DSM criteria, it is important to consider individuals symptomatic adult ADHD due to the changes made in the diagnostic criteria over the years.

## 2 Clinical presentation

The etiology of ADHD highlights three impaired fundamental functions that affect individuals with ADHD at both cognitive and behavioral levels: inattention, hyperactivity and impulsivity. Attention refers to a set of both executive and non-executive functions such as alertness, the ability to select information and signal processing and it operates across both perceptual and non-perceptual domains (Oberauer, 2019). Impaired attention is one of the key indicators of ADHD along with poor planning, listening, organization and concentration abilities and distractibility. For individuals with ADHD, sustaining attention may be challenging, although they could easily hyperfocus on activities which they may find interesting (Bijlenga et al., 2019). Similarly, they exhibit reduced divided and selective attention compared to neurotypical individuals (Tucha et al., 2017). Hyperactivity is defined as excessive verbal behavior and physical movement (Sarver et al., 2015). In children with ADHD, hyperactivity presents itself with behaviors such as being impatient for waiting turn, interrupting conversations, fidgeting and excessive physical activity. Whereas in adults, hyperactivity may present itself differently such as exercising excessively and inability to relax or sleep (Bijlenga et al., 2019). Lastly, impulsivity is characterized by complex factors such as sensation-seeking, difficulties in delaying gratification and acting with limited forethought, which are also present in both internalizing and externalizing disorders (Johnson et al., 2017).

Beyond these three established symptom domains, nuanced impairments in executive functions (EF) are observed in ADHD (Pineda-Alhucema et al., 2018). EFs are characterized as the set of skills such as planning, reasoning, exhibiting goal-directed behavior, monitoring, sustained attention and problem-solving. These neurocognitive abilities are impaired in the ADHD pathophysiology and are common across all ages in ADHD due to delayed fronto-cerebral network development. Additionally, they are more pronounced in children and adolescents due to the ongoing maturation of the brain, with greater impairments observed among those with comorbidities (Sadozai et al., 2024).

According to the unifying theory of ADHD established by Barkley (1997), EF dysfunction in ADHD is evident across four domains: behavioral inhibition, working memory, internalized speech, and the regulation of emotions, motivation, and arousal. These functions interact to support goal-directed behavior and the prefrontal cortex has a key role in coordinating and integrating these processes. The model highlights that EFs are not just about impulse inhibition but also about generating new responses to challenges, making decisions, and adapting to complex situations, implying cognitive flexibility and self-regulation.

Working memory deficits which are responsible for storing and processing information, are frequently associated with poor organizational skills in children with ADHD aged 8–13 (Kofler et al., 2018). These impairments may decrease with age due to brain maturity, though attention difficulties can also interfere with working memory performance (Ramos et al., 2020). Moreover, impairments in domain-general central executive working memory, rather than its individual subcomponents, have been linked to ADHD severity, indicating that broader working memory mechanisms may significantly influence ADHD symptoms (Fosco et al., 2020). Inhibitory control, which is also critical for self-regulation, is a central feature of deficits in ADHD, as highlighted by Barkley’s EF theory (Barkley, 2025). Barkley argues that response inhibition is a central aspect of EF, and that it is essential for the adequate functioning of EFs. On the other hand, the ability to develop strategies, problem-solving and decision-making are considered as part of cognitive flexibility. In particular, the ability of task-switching and goal-directed behavior are determined by the level of cognitive flexibility. Compared to healthy controls, cognitive flexibility is considerably lower in those with ADHD (Roshani et al., 2020).

Following Mahone and Denckla’s debate on early neuropsychological theories of ADHD based their considerations on disturbances in EF (Mahone and Denckla, 2017), the most integrated model by Barkley (1997) indicates that behavioral inhibition to be a core impairment that consequently gives rise to difficulties with working memory, self-regulation, and motor control. The authors follow by explaining more recent theories have expanded this notion by incorporating additional concepts such as state regulation, delay aversion, and response variability. And most importantly, they underline the dynamic heterogeneity of ADHD, as stated in the dual-pathway model (Sonuga-Barke, 2003). This model links executive dysfunction and reward processes, particularly delay aversion. It specifies that ADHD is caused by dysregulation in two interconnected pathways: the executive circuit and the reward circuit. Dysregulation of the executive circuit, especially inhibitory control, causes dysfunction in the EF and negatively influences self-regulation and decision-making skills. Concurrently, abnormal functioning of the reward circuit results in delay aversion. Delay aversion, also known as temporal discounting, refers to the preference for immediate over delayed rewards, often driven by dopaminergic dysregulation (Mahone and Denckla, 2017; Kanarik et al., 2022). This is highly pronounced in ADHD and sheds light on the tendency to form maladaptive habits, such as smoking, gambling or impulsive spending, which all provide instant gratification (Weinsztok et al., 2021). Conversely, if they experience motivational challenges (e.g., performing long or not stimulating tasks), this may also exacerbate the ADHD symptoms (Posner et al., 2020). These frameworks explain how impulsivity and impaired self-regulation contribute to ADHD-related negative outcomes (Sonuga-Barke, 2003; Shen et al., 2020). Nevertheless, it should be noted that the impact of executive dysfunctions on daily functioning may also vary significantly among individuals with ADHD and that many factors such as presence of secondary psychiatric conditions and age may contribute negatively to these impairments (Crisci et al., 2021).

## 3 DSM diagnostic criteria and classification

### 3.1 DSM over the years

Acknowledgement of ADHD reaches back to 1775 in medical literature (Faraone et al., 2021). Nevertheless, there has been debate over the years regarding its classification system. As a result of the discrepancies, the diagnostic criteria have undergone revisions multiple times. Updates in the DSM manual for the diagnostic criteria for ADHD reflect all the improvements and advancements regarding understanding this disorder.

The diagnostic value of ADHD first took place in 1968 when it was named as the “Hyperkinetic Reaction of Childhood in DSM-II (American Psychiatric Association, 1968). As per symptoms of the disorder, hyperactivity, short attention span, restlessness, and distractibility were included in the criteria, much as they are in the current description of ADHD (Barkley, 2011). In 1980, the focus on the symptoms shifted from hyperactivity to inattention. ADHD gained popularity as inattention was discovered to be another symptom. DSM-III (American Psychiatic Association, 1980) reclassified ADHD under two divisions: ADD with hyperactivity and ADD without hyperactivity. In 1987, hyperactivity was reintroduced as a core symptom in the revised version of DSM (DSM III-R) (American Psychiatric Association, 1987). DSM III-R defined the disorder with the symptoms (inattention, hyperactivity and combined) all together without any subtypes. Additionally, ADHD was titled as Disruptive Behavior Disorder (DBD). Later in 1994, DSM-IV (American Psychiatric Association, 1994) brought back the subtypes as predominantly inattentive, predominantly hyperactive and combined. This version slightly broadened the definition of ADHD by incorporating instances related to social, vocational, and academic settings in the diagnostic criteria, implying that ADHD was accepted to be not just a disorder affecting children. The revised version of DSM-IV (DSM-IV-R) (American Psychiatric Association, 2000) was later released in 2000 and a fourth sub-category named “not otherwise specified” was added. The impairment had to be present before the age of 7.

### 3.2 DSM-5 criteria for ADHD

DSM-5 was published by the American Psychiatric Association in May 2013 (Steinau, 2013; Surís et al., 2016). DSM-5 is the latest edition of the DSM and replaces the DSM- IV-TR published in 2000. Although new disorders were generally included in DSM-5, some changes were made such as changing the diagnostic criteria of some existing disorders (American Psychiatric Association, 2013; Kendler, 1017). As the latest updated version of the DSM, DSM-5 criteria require that symptoms exacerbate or degrade the quality of social, vocational, and intellectual functioning and affect two or more areas of daily functioning to diagnose an individual (American Psychiatric Association, 2013). Clinically, ADHD involves three presentations: inattention (ADHD-I), hyperactive/impulsive (ADHD-HI) and combined (ADHD-C). Inattention presentation includes the inability to pay or sustain attention, impaired thinking, inability to finish tasks, disorganized, distracted easily, and forgetfulness. The hyperactive/impulsive presentation includes the following symptoms: fidgeting, excessive talking, frequently interrupting, excessive physical activity, and inability to wait or take turns. Individuals who exhibit both inattention and hyperactivity/impulsivity symptoms are classified under the ADHD combined presentation (Rigler et al., 2016). In the previous versions, these presentations were referred to as “subtypes.” However, this terminology has been updated as “presentations” in DSM-5 in order to reflect that the symptoms are not stable and may change over time (Epstein and Loren, 2013; Leffa et al., 2022).

Considering all the developments in the history of ADHD, the DSM-5 was issued in 2013 with significant changes in the criteria. ADHD has become recognized as a neurodevelopmental condition rather than Disruptive Behavior Disorder in the DSM-5. ADHD symptoms are classified as severe, moderate, or mild, with more flexibility regarding the absence or presence of symptoms in social, occupational or academic settings (Steinau, 2013; Surís et al., 2016). While symptom changes may be observed due to maturity, the DSM-5 notes that problems with hyperactivity, poor attention and impulse control persist and that many young individuals with ADHD persist substantially impaired even throughout adulthood. According to the symptomology presentation indicated by DSM-5, the criteria suggest that the individual should be experiencing (1) inattention or (2) hyperactivity and impulsivity or both patterns together for at least before the age of 12 without any psychotic disorder background. These symptoms must be present in two or more settings (social, academic and occupational life), resulting in impairment (American Psychiatric Association, 2013).

Some of the changes in the DSM have caused concerns from a clinical point of view. These changes have paved the way for adult diagnosis (Young and Goodman, 2016). For ADHD diagnosis in adults, DSM-IV required the presence of at least six symptoms, while DSM-5 reduced that criterion to five for adults (Maltezos et al., 2020). The reduction of the ADHD cut-off points in DSM-5 compared to DSM-IV has increased the likelihood of receiving the diagnosis of ADHD. Nevertheless, both versions include 18 items in total, the first nine items are related to inattention and the other nine items address hyperactivity and impulsivity symptoms. However, unlike DSM-IV, the ADHD criteria of DSM- 5 are no longer only for children. On one hand, this may be recognized as an ethical problem because the cut-off scores for the diagnostic criteria have been reduced. On the other hand, it has enabled many people to receive the diagnosis, access the adequate intervention and thereby improve their quality of life. Thus, the changes to the DSM-5 criteria for ADHD more accurately reflect the current scientific understanding of the disorder and help to better capture the diversity of symptoms and impairments experienced by individuals with ADHD.

While the DSM-5 introduced several changes, it is still recommended to consider factors such as age of onset and areas affected by symptoms (Rigler et al., 2016). Age of onset of ADHD is one of the most discussed concepts in the DSM. Debate over whether ADHD is an early age-onset or “late-onset” is still being debated (Caye et al., 2017; Cooper et al., 2018). The concept of late-onset does not emphasize the later onset of the disease, but the manifestation of the symptoms later in life. Some individuals with ADHD may compensate the cognitive and behavioral impairments by developing coping strategies in childhood or it could be masked or misdiagnosed by other conditions such as anxiety or depression (Riglin et al., 2022; Onandia-Hinchado et al., 2021). In such cases, not only clinical presentation but also socioeconomic and cultural differences are of great importance.

The previous versions of DSM involved statements oriented to children and adolescents. However, DSM-5 contains more general statements relevant to all age groups. For instance, in the DSM-IV, the statement “often loses things necessary for task activities” applied to toys, school-related activities or items. In DSM-5, these examples were expanded to items which also apply to adults (e.g., wallets, paperwork, keys). Compared to DSM-IV, DSM-5 has allowed the diagnostic utility for adult screening to some extent. In the previous version of DSM, while the diagnostic criterion age was seven, this was increased to 12 in DSM-5. While for those younger than 17 years of age six symptoms from either domain is considered sufficient, for those older than 17 years of age, this criterion is reduced to five. Hence, the change in age of onset is also one of the factors that facilitates the diagnosis of adults (Posner et al., 2020).

### 3.3 Diagnosing ADHD

Diagnosing ADHD requires a comprehensive assessment that considers both functional and cognitive impairments. It is essential to consider how ADHD affects an individual’s daily life across multiple areas, including academic performance, social relationships, and occupational functioning, rather than focusing solely on meeting the diagnostic criteria (Posner et al., 2020). Cognitive impairments, on the other hand, are important for predicting functional outcomes, understanding symptom presentation and differential diagnosis. Assessing the both of these impairments enables clinicians to develop tailored interventions and pinpoint the specific areas that require special attention.

Clinical interviews conducted by healthcare professionals have been the most acknowledged means of screening to establish an appropriate diagnosis (Danielson et al., 2018). It is substantial to determine the severity, duration, outcomes, genetic predispositions and the potential existence of any co-occurring conditions and ensure that the symptoms match the criteria and do not belong to another disorder. Apart from interviews, clinical experts often conduct multiple methods such as neuropsychological and behavioral assessment tools as well as gathering information from other informants involved in the patient’s life (NICE, 2018; Hall et al., 2016). However, to ensure interview quality, clinical interviews should be conducted first followed by secondary screening tools.

#### 3.3.1 Diagnosing child and adolescent ADHD

In recent years, the recognition of ADHD has greatly improved, especially in child ADHD by family members and educators. This increased awareness has led to a greater number of individuals seeking attention and intervention from professionals, including psychologists, psychiatrists and general practitioners (McGough, 2014). About one in eight children with ADHD receive intervention from a psychologist, while most children with ADHD are cared for by a physician (French et al., 2020). According to National Institute for Health and Care Excellence (NICE), recommended steps for children and adolescent ADHD is through pediatric services where the diagnosis is established by child and adolescent psychiatrists (Danielson et al., 2018). Initial diagnosis of ADHD requires several important types of assessment. These include the child’s family history and a thorough medical evaluation (e.g., neurological examination, laboratory tests). This is because there may be possible pathological conditions that may resemble ADHD. However, a child or adolescent diagnosed with ADHD may also have other disorders that also require further evaluation (Wolraich et al., 2019).

In preschool children, diagnosing ADHD may be challenging due to the overlap with typical distractibility and hyperactivity for their age. Children with ADHD often show excessive hyperactivity and impulsivity, along with difficulties such as sleep disturbances, tantrums, and aggression. Severe cases may persist over time, but early symptoms may improve as the child ages (Hall et al., 2016). For school-aged children, diagnosis largely relies on reports from parents and teachers, although self-report is also considered. This is because hyperactivity is often observable in classroom settings through behaviors such as restlessness, chattering, and difficulty sitting still in class, while the indicators of ADHD-I include challenges such as difficulty focusing, forgetting homework, and losing materials. These issues are often accompanied by social and behavioral problems (Cuffe et al., 2020). Observing the child in natural settings, such as classrooms, provides additional information about ADHD-related behaviors. In this period, common comorbidities could be observed.

An initial diagnosis of ADHD is also common in adolescence, usually due to the emergence of difficulties in the school, social and home environments, and the possible involvement in impulsive or risky behaviors. Adolescent ADHD often includes engagement with risky behaviors, which can lead to negative outcomes such as substance abuse, academic withdrawal, and mental health challenges like suicidal thoughts. External factors like family, peer relationships, and social circumstances further influence symptom severity and associated risks (Eccleston et al., 2019). Diagnosis is usually based on adolescent self-report, with parental and teacher input. Symptoms can vary in this age group, with adolescents reporting inner restlessness, motivation and organizational difficulties (Hall et al., 2016). Comorbidities such as depression, anxiety and substance use disorders may be more common, particularly during significant developmental transitions such as the pubertal period. Therefore, a more comprehensive approach may be required during this period compared to childhood diagnoses.

For child and adolescent assessments, clinical interviews both with the child and a secondary respondent is always strongly recommended. Specifically in the rating scales completed by teachers, respondent bias could be observed in case of very young children compared to older children (Hall et al., 2016). Hence, both objective and subjective measures should be gathered by parents and teachers in order to avoid conflicting information arising from potential biases or differing level of involvement. This would also allow the clinician to obtain broad information regarding the functional and cognitive impairments in multiple settings. By interviewing these secondary respondents, wider perspective of the child’s clinical presentation such as finding out when, in which settings and how the symptoms arise, the child’s developmental history, daily life and relationships with others and the settings where impairments manifest the most (e.g., unable to sit still in the classroom, unable to pay attention to activities) could be obtained. That is why it is commonly advised to access school records, academic performance, and medical history in ADHD diagnosis for children and adolescents. Therefore, in addition to parent interviews, it is advisable to gather information from teachers regarding the child or the adolescent’s behavior in more than one setting alone (NICE, 2018).

There are challenging parts of clinical interviews in pediatric diagnosis (Hall et al., 2016). One of these challenges is that children, unlike adolescents and adults, have lower verbal competences. Therefore, they may not be sufficiently expressive in clinical interviews. Families may be resistant to medication or other interventions to prevent their children from being stigmatized. In teacher interviews, the reporter bias may be high because the child behavior may differ from home to school environment, however it may be able to detect the ADHD-HI or ADHD-C presentation rather well. Besides, in the early ages, potential issues such as social and intellectual maturation, psychological state, or health conditions must be addressed. While working with children, if feasible, it is recommended to discuss their condition with their parents, teachers or any other important figures in their lives to obtain a better understanding of the child (Geddes and Andreasen, 2020).

#### 3.3.2 Diagnosing adult ADHD

ADHD was long believed to be a childhood disorder that children grow out of as they age. However, many issues such as inattention, poor impulse control, subjective restlessness, poor planning, disorganization, poor self-regulation, and other deficits are highly likely to persist into adulthood (Slobodin et al., 2018). Due to that, diagnosis in later in life is also prevalent and common in ADHD and under-diagnosed or misdiagnosed cases of adult ADHD are highly common (Ginsberg et al., 2014).

Adults face significant psychological, social, and academic stressors, which can adversely affect their EF and emotional regulation. These challenges along with poor self-regulation later extends to occupational settings (Harpin et al., 2016). While hyperactivity is physically manifested in childhood, in adulthood it tends to be more internalized. It often appears as risky behaviors, financial difficulties, or restlessness (Anbarasan et al., 2020) accompanied by EF impairments. In older adults, there is limited ADHD research available which could be partly due to the challenges coming along with cognitive decline and behavioral issues associated with it. Co-occurring conditions like anxiety and depression may further complicate the diagnosis (Kooij et al., 2016) and traumatic or stressful experiences could also resemble ADHD-like symptoms (Marshall et al., 2021).

Age-appropriate symptom profile for adult ADHD in line with the DSM-5, the criteria for adult ADHD should be judged very differently from the criteria for child and adolescent ADHD (Maltezos et al., 2020; Asherson et al., 2016). When considering the symptoms of inattention, the behaviors that we may encounter in adult ADHD include distraction while talking and performing a task, inability to finish initiated tasks or projects, failure of performing individual responsibilities (e.g., paying taxes or bills, doing house chores), forgetfulness, vocational issues and inability to manage time. On the other hand, hyperactivity is seen in a very distinct form than in childhood. In adults with ADHD-HI or ADHD-C presentations, behaviors such as inability to sit for long periods of time, having the urge to move often, interrupting others during conversations and excessive talking are highly common (Gentile et al., 2006).

National Institute for Health and Care Excellence (NICE) (Danielson et al., 2018) guidelines suggest that adults without a formal childhood diagnosis presenting with ADHD symptoms should be referred to adult specialist services. These services assess the typical features of ADHD that emerge in childhood and persist into adulthood, the possible co-existence of other psychiatric conditions, and the identification of significant psychological, occupational or social impairment. Also, behavioral and functional impairments related to EF should be carefully assessed as previously suggested by Brown’s EF model of ADHD (Brown, 2008). These functional areas include activation, focus, effort, emotional, memory and action. These domains are specifically sensitive to cognitive and behavioral diagnostic tools.

Late-onset ADHD is also widely discussed in the literature. Caye et al. (2017) highlights that this phenomenon, also referred to as “*de novo* adults” is highly common in adults where a significant proportion of them do not meet the ADHD criteria in childhood but prevalence rate of late-onset adult ADHD is 4.4%. They explain this through two models: complex phenotype model, and the restricted phenotype model. Complex phenotype model suggests that biological and environmental factors could lead to the emergence of symptom later in life. ADHD symptoms may remain dormant during the early ages or compensated via protective factors such as positive family environment. However, the transition from childhood to adulthood also implicates higher environmental demands which may contribute to the visibility and the aggregation of the symptoms. Whereas the restricted phenotype model argues that other pre-existing conditions such as trauma, substance abuse, hormonal imbalances may be the main cause of ADHD-like symptoms. Determining whether these late-onset symptoms are due to true ADHD cases requires a multimodal approach where behavioral and cognitive indicators, medical history and environmental stressors are explored through assessment. On the other hand, the environmental stressors may aggregate or reduce the symptoms resulting in symptom fluidity. The severity of the symptoms can fluctuate over time as well as the symptom presentations. Inattention is most likely to persist throughout life, while hyperactivity and impulsivity tend to decrease more over time (Emser et al., 2018). However, the neuropsychological deficits such as attentional vigilance and EF start in childhood and could continue to impact everyday life, persisting into adulthood (Moffitt et al., 2015).

## 4 Evaluation tools

### 4.1 Clinical interviews

Clinical interviews have always been of greater clinical value than neuropsychological testing, computerized tests, standardized questionnaires and other tools (Hall et al., 2016). They are divided as structured and unstructured. Structured interviews are objective with strictly predefined questions. Unstructured interviews are performed with a sequence of questions asked by the interviewer that are unpremeditated and adjusted based on the course of the session (Marshall et al., 2021). Although they may help the interviewer discover different aspects and the needs of the patient and build comprehensive rapport, they are low in reliability, more subjective and depend on the expertise level of the interviewer. Consequently, structured interviews provide a systematic and standardized framework for clinicians. These interviews help the clinicians obtain the information that rating scales may not uncover, as structured interviews assess the presence of disorders and comorbidities.

A model based on the DSM has been proposed by a previous study, suggesting a 5-point model to facilitate a more accurate assessment within the diagnostic system, consequently enhancing clinical utility (Chang et al., 2016). Firstly, it is argued that assessing how mental health disorders are initially conceptualized is crucial for determining their clinical utility. Secondly, there is emphasis on the importance of the accurate communication of the relevant clinical information from the relevant parties (e.g., patient and patient’s family, practitioners, healthcare professionals). Thirdly, it is reported that the diagnostic criteria should always be used in the clinical interview. Lastly, the right interventions for the patient should be identified, and the patient should be guided adequately considering their current and future needs. Similarly, there are other important aspects in a clinical interview (Barkley, 2011). To obtain a diagnosis of ADHD, firstly, the patient who comes to the consultation must be questioned about any experienced symptoms in childhood and present symptom complaints. Further approach for establishing a diagnosis could be taken by conducting a formal interview with someone close to the patient (e.g., a family member, friend, teacher or partner) to further gain information about their daily functioning and social relationships in addition to behavioral observation, academic or medical reports (Posner et al., 2020). However, the interviewer is also an important determinant for the quality and the variety of the screening. Objective measurement and additional screening tools such as rating scales, computerized tests and many other tools that may support the accuracy of the diagnosis are of great importance (Hall et al., 2016).

#### 4.1.1 Which tools are commonly used?

The Diagnostic Interview Schedule for Children (DISC-V) (Shaffer et al., 2000) is a structured interview DSM-IV based screening tool. It involves six modules and approximately 3,000 questions. From these, 358 of them are categorized as stem questions meaning that they are standard questions given to all respondents. Around 1,300 contingent questions are asked to avoid false positive responses directed during stem questions. The information on age of onset is highly considered to determine the clinical standpoint and the duration of the symptoms. To ascertain the level of impairment, the respondents are also asked questions which involve multiple social settings, as stated in the diagnostic criteria. Additionally, questions regarding the medical history and the family history are also included.

The Young DIVA-5^1^ is a structured clinical interview designed to assess ADHD symptoms in children and adolescents aged 5–17 years. Previous versions have shown high diagnostic accuracy and practical use among adults (Ramos-Quiroga et al., 2019). It is based on the DSM-5 criteria, and DIVA for a comprehensive evaluation of ADHD symptoms across different age groups. The Young DIVA-5 adapted to the developmental and behavioral factors specific to children and adolescents. Although there is limited evidence on the Young DIVA-5, the high potential demonstrated by earlier versions suggests that it could also be an ideal tool for assessing ADHD in younger populations.

In adult clinical diagnosis, similar tools are generally used. One of the most frequently used tools is The Diagnostic Interview for ADHD in Adults (DIVA) (see text footnote 1) (Kooij and Francken, 2010) which also includes a version for children adolescents. The DIVA was first introduced in Dutch in 2007 and recently it has been updated according to the DSM-5 and translated into more than 25 languages. It has three main sections as follows: (1) childhood and adulthood symptoms, (2) the age of onset of symptoms and (3) areas of impairment (Kooij, 2022). There are 18 DSM-oriented items in total and this semi-structured interview involves retrospective questions (about the childhood). Moreover, DIVA is also offered for free and online via an app, which facilitates the access which means that it also helps the individual to do a self-assessment for suspected ADHD. Given that it is a relatively recent version, there has not been extensive work on the clinical utility of this interview tool. The previous versions such as DIVA-2 was revealed to detect ADHD more accurately than neuropsychological testing and rating scales (Pettersson et al., 2018). Nevertheless, studies conducted with both Korean and Persian samples in DIVA-5 show high sensitivity (SN) and specificity (SP) (Hong et al., 2020) and good test–retest and inter-rater reliability (Zamani et al., 2021). These studies show that DIVA-5 is a reliable tool and can discriminate individuals with ADHD from control subjects accurately. Furthermore, Conners Adult ADHD Diagnostic Interview for DSM-IV (CAADID) is a gold standard DSM-based semi-structure tool and is used in the diagnosis of ADHD in both children and adults (Ramos-Quiroga et al., 2019; Epstein and Kollins, 2006). CAADID’s ability to distinguish healthy controls from adults with ADHD and that it has good test–retest reliability.

To summarize in general, clinical interviews are considered as the gold standard for ADHD diagnosis. However, to accurately diagnose ADHD, firstly, a clinical interview based on the diagnostic manual is required. It is crucial to use one or more assessment tools, and to obtain a report from an informant involved in the individual’s life to assess the individual. The reason for this is that ADHD criteria may vary from children and adolescents to adults and functional and cognitive impairments may change with age (Matte et al., 2015). However, its crucial to recognize that there is limited research regarding the accuracy of the clinical interviews (Marshall et al., 2021). Although there are tools with promising psychometric values, it is important to employ a multifaceted approach to ensure an accurate ADHD evaluation. The inclusion of structured DSM- based interviews and other screening instruments can provide a clearer and more reliable profile of the patient’s condition. The use of multiple diagnostic tools is necessary to create a more accurate and reliable ADHD diagnostic system.

### 4.2 Use of rating scales in the assessment of ADHD

In addition to clinical diagnosis, rating scales have a very important part in diagnosis of ADHD. Rating scales help the clinician to validate the presence of the disorder and to identify possible co-existing conditions. In addition, availability of multiple respondents in some rating scales allows the clinician to evaluate the patient profile with the responses of various people (e.g., family members, teachers). They are also a useful tool that allow the clinician to choose the most suitable treatment approach.

Rating scales are often the most favored evaluation tool after clinical interviews. Although they are secondary, they play a crucial role in addressing the limitations of clinical interviews. In clinical interviews, if the patient is young, the primary interviewee is usually a parent or guardian, although the patient is still interviewed. Conversely, the parents may provide broad perspective, however their impartiality should be considered. Similarly, children’s limited ability to express themselves emotionally and verbally may influence the clinician’s judgment. And in adolescents, they may also exhibit inconsistencies in self-report or deny experiencing problems. This situation highlights the importance of using rating scales with informants as a secondary diagnostic tool in the assessment of ADHD.

From a clinical standpoint, numerous approaches have been developed to evaluate the quality and usefulness of a tool, and several models have been proposed for it. Numerous factors are reported in the literature to be associated with clinical utility (Smart, 2006). These are usually related to many factors such as duration, accessibility, assessment, and the pricing of the tools. According to a previously suggested model, there are six elements to determine clinical utility of the rating scales. These are as follows respectively: (a) ease of use, (b) time, (c) training and qualifications, (d) format, (e) interpretation, and (f) meaning and relevance of information obtained. In general, the criteria considered here are the availability of the tool, its price, the clarity of the language of the instructions given for both the clinician and the patient, the time given and the effect of this time on reliability and validity, the acceptability of the tool for both the patient and the clinician, the expertise of the clinician and the requirements for interpreting the results, and many other minor criteria are encompassed in these six elements. Similarly, a multi-dimensional model indicates that there are four main elements to determine clinical utility: appropriateness, accessibility, practicability, and the acceptability (Smart, 2006). With these four elements, several important factors such as the appropriateness of the tool in general, its clinical significance, price, accessibility, whether it would cause ethical, legal, social or psychological concerns.

Rating scales are valuable in diagnosis and management, having been rigorously evaluated using psychometric indices such as SN, SP predictive power, and area under the curve (AUC) (Conners, 1999). SN is defined as the capacity of a test to detect patients who have illness/disorder which could be thought as percentage of the cases. SP shows how well the test can distinguish between different groups (e.g., ADHD vs. non-ADHD). In addition, AUC is also important in determining the quality of the diagnostic test. AUC value between 0.90 and 1.00 is considered as above excellent, between 0.80 and 0.90 as good, between 0.70 and 0.80 as fair, between 0.60 and 0.70 as poor, and between 0.50 and 0.60 as unsuccessful (Florkowski, 2008).

Use of rating scales involve both advantages and drawbacks. One possible advantage of rating scales is that they are uncomplicated and inexpensive to administer compared to clinical and neuropsychological testing tools. They could be applied in a variety of settings. They aid in screening and diagnosis procedure, the identification and measurement of the target symptoms and behaviors, treatment outcome, the frequency and severity of ADHD symptoms (Krieger and Amador-Campos, 2018). Similarly, rating scales are quick to administer and economically convenient (Rogers et al., 2022) although, they do not provide an elaborative diagnostic value as clinical interviews do. However, one of the issues with the standardized measures is that many studies focus on assessing ADHD by only utilizing rating scales based on threshold values to diagnose patients instead of using gold-standard clinical interviews (Mulraney et al., 2021). Compared to clinical interviews, rating scales are limited in terms of the variety of statement. Due to that, limiting the diagnosis to rating scales only could lead to misdiagnosis. In some cases, rating scales are more efficient at covering symptoms or potential problems than clinical interview tools except it is often achieved in less profundity. This is because rating scales do not always include information about the onset of the disorder, the duration of its existence or the relevant factors that may be contributing to the symptoms. Additionally, respondent bias in rating scales also may be involved (Döpfner et al., 2006).

Although the psychometric properties of a rating scale may be good, it is still a limited tool compared to clinical interviews. In general, rating scales should be considered secondary and used when significant suspicion of ADHD is present due to their limited ability to identify accurately (Chamberlain et al., 2021). Nevertheless, understanding an individual’s specific behavioral and EF challenges in everyday settings identified through rating scales could help clinicians tailor interventions to address those needs directly (Krieger and Amador-Campos, 2018).

#### 4.2.1 Specific rating scales used for child and adolescent ADHD

There are several rating scales that are frequently used in the measurement of child and adolescent ADHD. Scales such as Conners, ASEBA scales, Vanderbilt and Swanson, Nolan, and Pelham (SWAN), Strengths and Difficulties Questionnaire (SDQ) are highly relied on especially for observing treatment response such as side effects or behavioral changes (Kemper et al., 2018). The most frequently used rating scales in children and adolescents are the following:

These rating scales are provided in Table 1 including detailed information along with a summary of their psychometric properties reported by empirical studies and systematic review and meta-analyses. Important values such as SN, SP and AUC are primarily reported. When these values were unavailable, other reliability and validity metrics were included where possible.

**Table 1:** Commonly used rating scales in child and adolescent ADHD.

Family	Scale name	Rater	Factors/Subscales	Psychometric properties	Source
Achenbach System of Empirically Based Assessment (ASEBA) (Achenbach and Rescorla, 2014)	Child Behavior Checklist (CBCL)	Parent/caregiver (113 items)	Problem behavior scale (8 subscales) Social competence scale	CBCL/6–18: SN 81% SP 70% AUC (95% CI) 0.81 [0.75, 0.85]	Skarphedinsson et al. (2021)
	Teacher Report Form (TRF) (Achenbach, 1991a)	Teachers (113 items)	Affective Problems Anxiety Problems Somatic Problems Attention Deficit/Hyperactivity Problems Oppositional Defiant Problems Conduct Problems.	90% SN (T ≥ 54.8): SN 90% SP 27%	Jarrett et al. (2018)
	Youth Self Report Form (YSR) (Achenbach, 1991b)	Self-report (112 items)	Overall functioning sports (activities), mood, and anxiety (anxious/depressed, withdrawn/depressed, somatic complaints), general symptomatology (social problems, thought problems)	SN: 65% SP: 70% AUC 0.71 [0.63, 0.78]	Skarphedinsson et al. (2021)
Conners Rating Scales (Conners, 2024)	Conners 4-P Conners 4-T Conners 4-SR	Parent/caregiver Teacher Self-report	Content scales (Inattention/Executive Dysfunction, hyperactivity, impulsivity, emotional dysregulation, depressed mood, anxious thoughts) Impairment & functional outcome scales (schoolwork, peer interactions, family life) DSM Symptom scales (ADHD-I symptoms, ADHD hyperactive impulsive symptoms, total symptoms, ODD symptoms, CD symptoms) Conners 4-ADHD Index	NR	–
Swanson, Nolan and Pelham Teacher and Parent Rating Scale (SNAP-IV) (Bussing et al., 2008)	SNAP-IV (90-item) SNAP-IV (26 item)	Parent/caregiver and teacher (90 items)	Inattention Hyperactivity/impulsivity ODD symptoms	Based on DSM-5 ADHD criteria Parent rating scale: SE: 87% SP: 56.9% Teachers: SE: 90.6% SP: 31.3%	Hall et al. (2020)
The Strengths and Weaknesses Questionnaire (SDQ) (Goodman, 1997)	SDQ Parent (SDQ-P) SDQ Teacher (SDQ-T)	Parent/caregiver (25 items) Teacher (25 items)	Emotional problems, conduct problems, hyperactivity-inattention, peer problems, pro-social behavior, total difficulties	Clinical cut-off: SN 0.59 [0.46, 0.70] SP 0.79 [0.65, 0.89]	Pooled results (Mulraney et al., 2021)
Vanderbilt ADHD Diagnostic Scales (Wolraich et al., 2003)	Vanderbilt ADHD Diagnostic Scales (VADPRS) (WOLRAICH et al., 1998) The Vanderbilt ADHD Diagnostic Teachers Scale (VADTRS) (Wolraich et al., 2003)	Parent/caregiver (45 items) Teacher (35 items)	18 items corresponding to ADHD symptoms: peer relations, disrupting class, assignment completion, organizational skills, written expression, mathematics, reading	Cronbach’s *α* values: 0.94 (total ADHD) 0.92 (ADHD-I) 0.91 (ADHD-HI)	Anderson et al. (2022)

The rating scales used to diagnose and assess ADHD (see Table 1) are valuable tools for gathering information from multiple respondents (parent, teacher, self-report). The Child Behavior Checklist (CBCL) shows high SN (81%) and moderate SP (70%), with AUC of 0.81, suggesting its utility in identifying ADHD in children. The Teacher Report Form (TRF) is SN (90%) but has lower SP (27%). Similarly SNAP-IV scale yields high SN rates ranging from 87% (parent report) to 90.6% (teacher report), but the SP values are considerably lower. The Vanderbilt ADHD Diagnostic Scales (VADS), shows high reliability, with high reliability values of 0.94 for total ADHD symptoms. On the other hand, limited information exists regarding Conners 4 rating scales, as this version is relatively new.

Overall, while the rating scales are valuable in clinical practice, their psychometric properties may be influenced by factors such as age, ADHD presentation, experience and training of the person administering the test, and the context in which the test is administered.

#### 4.2.2 Specific rating scales used for adult ADHD

The following is an overview of the most commonly used rating scales for the screening of ADHD in adults (see Table 2). The adult ADHD rating scales reviewed below showed variability in psychometric utility, with the Adult ADHD Self-Report Scale (ASRS-v1.1) showing the highest SN (0.90) and SP (0.88), making it an excellent screening tool (Kessler et al., 2005). The Barkley Adult ADHD Rating Scale-IV (BAARS-IV) and Conners’ Adult ADHD Rating Scales (CAARS) provide robust internal consistency and are reliable for assessing both childhood and current symptoms, although their test–retest reliability varies. Overall, these scales are valuable tools for diagnosing ADHD in adults, with high AUC and psychometric reliability, although combining them with other diagnostic methods would help obtain improved accuracy.

**Table 2:** Commonly used rating scales in adult ADHD.

Scale	Forms and number of items	Factors/subscales	Psychometric properties	Source
Adult ADHD Self-Report Scale (ASRS-v1.1) (Kessler et al., 2005)	Full version (18 items) General population screen (6 items)	Inattentive and hyperactive–impulsive	Full version: SN: 0.90 SP: 0.88 AUC: 0.903 (95%CI: 0.886–0.920)	Brevik et al. (2020)
Barkley Adult ADHD Rating Scale-IV (BAARS-IV)	Childhood symptoms self-report (20 items) Current symptoms self-report (30 items)	Childhood inattention Childhood hyper/impulsivity Childhood Total ADHD score Current Inattention Current hyperactivity Current impulsivity Current ADHD score	Internal consistency: (*α* = 0.78 − 0.91) Test–retest reliability: (*r* = 0.66 − 0.76)	Barkley (2011)
Brown Attention Deficit Disorder Scale (BADDS) (Barkley, 2011)	Self-report (40 items)	5 factors: Activation Attention Effort Affect Memory	Cronbach’s α: 0.95 SN: 72% SP: 88%	Kakubo et al. (2018)
Conners’ Adult ADHD Rating Scales (CAARS)	Long version - Self-report l (66 items) Long version - Other-report (66 items) Short version Self-report (26 items) Short version - Other-report (26 items)	Long version 9 factors: Inattention/Memory Problems Hyperactivity/Restlessness Impulsivity/ Emotional lability Problems with self-concept ADHD symptoms total ADH Index Inconsistency index Hyperactive–impulsive symptoms	Internal consistency: ranging from 0.49 to 0.97 (subscales and total scores)	Smyth and Meier (2019)
Wender Utah Rating Scale (WURS) (Ward et al., 1993)	Self-report (61 items) Short version (25 items)	Long version – 5 factors Learning problems, conduct problems, attention problems, social skills, stress intolerance Short version (3 factors) Impulsivity & Behavioral problems Impulsivity/Temper, Inattentiveness, and Mood/Self-esteem	Short version: Discriminating ADHD from controls SN: 91%, SP: 92% AUC: 0.974 Discriminating ADHD from psychiatric controls SN: 84 & SP: 94% AUC: 0.995	Gift et al. (2021)
The Adult ADHD Scale (Turgay ADHD Scale) (Turgay, 1995)	Self-report (30 items)	Part 1: Attention Part 2: Hyperactivity/Impulsivity Part 3: Features and Problems Related to ADHD	Part 1 Cronbach’s α: 0.80 Part 2 Cronbach’s α: 0.80	Turgay (1995)

AUC, area under the curve; CI, confidence interval; SN, Sensitivity; SP, Specificity; NR, Not Reported.

#### 4.2.3 General scales

Beyond the tools specialized in ADHD assessment, three additional factors are worth noting: cognitive, intellectual and emotional components. The utilisation of several scales could facilitate the evaluation of these domains, where conventional tools may exhibit limitations. A wide variety of EF rating scales could also be used for assessing everyday functioning in various domains of life. Among these scales, Behavior Rating Inventory of Executive Functions (BRIEF) (Gioia et al., 2000), the Behavior Assessment System for Children 3 (BASC-3) (Reynolds, 2010) could also provide valuable insight for many routes within the ADHD diagnostic and treatment planning. Furthermore, The Wechsler Adult Intelligence Scale (WAIS-V) (Weschler, 2024) Wechsler Intelligence Scale for Children (WISC-V) (Weschler, 2024) and Kaufman Brief Intelligence Test, Second Edition Revised (KBIT-2-R) (Kaufman and Kaufman, 2004) are often used in ADHD assessments because they evaluate a broad range of cognitive abilities, including verbal comprehension, perceptual reasoning, working memory, and processing speed. These tests do not measure ADHD directly, but they differentiate cognitive difficulties related to ADHD and other potential conditions, such as learning disabilities (LD) or intellectual impairments.

There are other scales that still could be valuable while not specifically targeting ADHD symptomology. Some scales may measure highly correlated constructs and, therefore indirectly measure ADHD. For example, the Self-vs. Externally-Regulated Behavior Scale, a recent scale (de la Fuente et al., 2022) measures self-regulation, dysregulation (such as hyper-response) and re-regulation behavior. Self-regulation, other than being related to emotional factors, is also intertwined with EF as previously indicated by Barkley (2025). In addition, Assessment System for Children and Adolescents (SENA) (Fernández-Pinto et al., 2015) addresses internalized behavioral problems and is one of the scales that can be used for a broader screening. The SENA has a multidimensional offering high reliability (0.70–0.80) that can be used in the initial diagnosis process for the age range of 3–18 years. It offers an assessment that covers areas such as emotional intelligence, awareness, competence, social integration and self-esteem.

Overall, these scales can be used to evaluate the heterogeneous nature of ADHD. And the use of general screening tests is essential for understanding the everyday functioning and identifying the cognitive impairments. This way, the clinicians could also provide a multimodal approach by screening the symptom fluctuations, identifying the functional and cognitive impairments. There is still a need for greater body of research regarding the clinical utility of the screening tools, including their SN, SP, and discriminant validity (Chang et al., 2016). The research and availability of these data is a necessary for understanding diagnostic value of the screening tools and their appropriate application in clinical practice and research.

### 4.3 Key neuropsychological tests and continuous performance tests

Up to this date, neuropsychological tests have been widely used to demonstrate EF deficits in ADHD. ADHD is linked to impairments in multiple cognitive domains, with notable deficits in EF such as behavioral inhibition, working memory, set-shifting, planning, and organization. These impairments may vary based on task context, with greater challenges observed during monotonous or lengthy activities compared to engaging ones.

Neuropsychological tests can help to identify specific areas of impairment in EF. A systematic review and meta-analysis has demonstrated that Children with ADHD exhibited larger delays in attention, response inhibition, planning, and working memory compared to children with tic disorders (TD) and specific learning disorders (SLD)s (Sadozai et al., 2024). However, while these measures aim to evaluate discrete EF domains, considerable overlap in the neurobiology underlying these domains challenges their functional independence. For example, the Wisconsin Card Sorting Test (WCST), typically used to assess set-shifting, may also depend on working memory processes (Sadozai et al., 2024).

Assessment tools for EF in ADHD typically fall into two categories: subjective and objective measures. Subjective tools include clinical psychiatric interviews and rating scales or questionnaires, often relying on reports from the individual or informants familiar with their childhood behaviors. Objective tools, on the other hand, involve neuropsychological tests and Continuous Performance Tests (CPTs), providing standardized and quantifiable data (Posner et al., 2020). Some of the commonly employed neuropsychological tools are provided in Table 3:

**Table 3:** Widely Utilized Neuropsychological Tests in ADHD assessment.

Test	Domain	Duration and age range	Parameters	Description and implications	Psychometric utility
Digit Span Task (Jones and Macken, 2015)	WM Attention	5-10 min 7+ years	Forward Span, Backward Span	Digit Span, a subtest of the Wechsler Intelligence Scale for Children (WISC-IV), assesses WM capacity involving sequences of digits in forward and backward order for recalling. While research suggests that WM impairments are a core feature of ADHD, a previous study revealed that performance on Digit Span can vary depending on the ADHD presentation and the individual’s age. ADHD group demonstrated poorer performance compared to the control group on both the forward (*t* = −3.12, *p* < 0.01) and backward digit span tasks (*t* = −2.83, *p* = 0.01) (Elosúa et al., 2017).	Digit Span might be sensitive to detecting WM difficulties in children with ADHD-C as well as differentiating ADHD presentation (Rosenbloom and Wultz, 2011).
D2 Test of Attention (Bates and Lemay, 2004)	Processing speed, selective attention and sustained attention	8-10 min 6 to 80 years	Omission errors, commission errors, processing speed, concentration performance, fluctuation rate and error distribution	The d2 Test of Attention offers a valuable assessment of selective and sustained attention and processing speed. The test requires the participants to identify the letter “d” while ignoring the other symbols. d2 test can be useful in distinguishing between ADHD presentations in children (Yato et al., 2019).	Cronbach’s α values (Krieger and Amador-Campos, 2021): Concentration performance: 0.74 Omission errors: 0.90 Commission errors: 0.61 Total errors: 0.90 Percent of errors: 0.90
Go/No-Go Task	Response inhibition Processing speed	10 min 3+ years	Omission errors, commission errors, reaction time, error rate	Go/No-Go task is used to assess processing speed and response inhibition. The task requires participant response to every “go” stimuli and withhold responses at “no-go” stimuli. Reaction time variability was identified as the only Go/No-Go performance measure that matched the discriminating capabilities of functional near-infrared spectroscopy (fNIRS) in differentiating children with ADHD group (children, 6–12 years) from healthy controls and commission errors significantly correlated with the fNIRS measures. However, the task alone did not correlate with symptom severity (Monden et al., 2015).	Sensitivity and specificity analysis (Li et al., 2016): SN: 0.967 SP: 0.833
N-Back Task (Braver et al., 1996)	WM	15-20 min7+ years	Omission errors Commission errors Perseveration False alarm	Between ADHD (20 children with ADHD -C, and 6 children with ADHD-I) and control group, no significant differences between groups were found for specific error types—omissions (*p* = 0.12), false alarms (*p* = 0.08), and perseverations (*p* = 0.81) (Elosúa et al., 2017).	Test–retest reliability (Elosúa et al., 2017) 0.65–0.92
Stop-Signal Reaction Time (SSRT) (Logan et al., 1984)	Inhibition	14 min 6+ years	Stop commission errors, go discrimination errors, go omission errors, go accuracy	SSRT is designed to measure response inhibition. No significant differences between ADHD and control group in SST measures. However, adults with ADHD made more omission errors and showed lower accuracy on “go” trials, suggesting difficulties with sustained attention alongside response inhibition (Senkowski et al., 2024).	Moderate effect size (Senkowski et al., 2024): Hedges’ *g* = 0.509–0.525
The Stroop Test (Stroop, 1935)	Attention, inhibition and cognitive flexibility	5-10 min 6+ years	Stroop Word-Color, Stroop-Color, Interference Score	The Stroop Test is frequently used to assess the inhibitory control and attentional deficits in individuals with ADHD. ADHD group (20 children with ADHD -C, and 6 children with ADHD-I) showed significantly worse performance than the control group on the overall task (*t* = −4.94, *p* = 0.01). No significant group differences were observed on the Stroop Word-Color (*t* = −1.81, *p* = 0.08), Stroop-Color (*t* = −1.72, *p* = 0.09), or Stroop Interference (*t* = 0.87, *p* = 0.39) (Elosúa et al., 2017).	Test–retest reliability (Elosúa et al., 2017): 0.71–0.98
Trail Making Test (Reitan, 1955)	Processing speed and cognitive flexibility	5-15 min 8 to 79 years	TMT-A (Part A): visual attention and task switching TMT-B (Part B): cognitive flexibility and executive function	ADHD group (20 children with ADHD -C, and 6 children with ADHD-I) required more time to complete Part B of the Trail Making Test, compared to the control group. No significant differences between the ADHD group and the control group in the time taken to complete Part A (t = 1.06; *p* = 0.29) Elosúa et al., 2017).	Test–retest reliability (Elosúa et al., 2017): 0.60-0.90
Tower of London (TOL) (Shallice, 1982)		10-15 min 7+ years	Task Analysis Cognitive Computations WM Inhibition of Impulsivity Stimulus-Bound Cognitive Flexibility Allocation and Maintenance of Attention	TOL evaluates planning ability, a key component of EF, by requiring participants to mentally plan and execute moves to achieve a target configuration. In an ADHD sample (56 children, 6–13 years), the task revealed deficits in goal-directed behavior and adaptive functioning (Unterrainer et al., 2020).	Cronbach’s α values (Unterrainer et al., 2020): glb: 0.83
Wisconsin Card Sorting Test (WCST) (Grant and Berg, 1948)	Cognitive flexibility, abstract reasoning, problem-solving and set-shifting	15-30 min 6,5 to 89 years	Total number of errors Perseverative responses Perseverative errors Non-perseverative errors Percent conceptual level response	WCST is widely used to evaluate EF, focusing on skills like set-shifting, behavioral self-regulation using external cues, and the tendency toward perseveration. Higher levels of inattention and overall ADHD symptoms among ADHD group (26 adults, aged 18 to 22) are significantly associated with reduced cognitive flexibility (Kercood et al., 2017).	Inconclusive results, mixed clinical sample (Cation and Schoenberg, 2024): 50.8% SN and 42.8% SP at cut-off of ≥1

glb, greatest lower bound of reliability; SN, Sensitivity; SP, Specificity; WM, Working Memory.

These widely utilized tests tests (see Table 3) assess different aspects of cognitive functioning such as attention, memory, inhibition and cognitive flexibility. The Go/No-Go task has high SN (96.7%) and SP (83.3%) for discriminating ADHD from healthy controls. Variability in other on tests such as the Stroop or WCST is often influenced by ADHD presentation, age and other individual factors. This may limit their generalizability across different presentations of ADHD. As a result, the effectiveness of the tests in the diagnosis of ADHD may be variable and they may not always reflect the complexity of the cognitive profile of ADHD.

One important limitation is that ADHD encompasses a range of cognitive profiles, with some individuals exhibiting deficits in EF, while others show difficulties in non-executive processes like memory, temporal processing, and motivation regulation (Barkley, 2025). Conversely, although EF impairments within the clinical presentation of ADHD have been widely implicated, it is critical to recognize that there may be individual variability in the levels of cognitive performance deficits. Recent systematic review has revealed that several studies support this variability; some studies report minimal EF differences in ADHD patients compared to controls, while others emphasize significant differences which could be due to the discrepancies between methods used (self-report and objective measures) for measuring EF (Onandia-Hinchado et al., 2021). This contrast underlines the complexity of diagnosing and assessing EF impairments in ADHD, as both subjective and objective measures can provide valuable but different perspectives. Additionally, it is essential to acknowledge the heterogeneous nature of ADHD. Cognitive impairments and their neurophysiological correlates may vary with age and the presence of comorbid disorders. Some struggle in many areas, while others have very specific problems and function well in other areas (Posner et al., 2020). This also highlights the need for further research to explore how the sensitivity of neuropsychological measures such as the N-Back or Go/No-Go task may vary in different ADHD subgroups (Breitling-Ziegler et al., 2020). Notably, neuropsychological tests alone are not sufficient to diagnose ADHD. A diagnosis should be made by a qualified professional using a multifaceted approach that incorporates various sources of information (Krieger and Amador-Campos, 2021).

Continuous Performance Tests (CPTs) are one of the complementary screening methods that are involved in the diagnosis of ADHD. The purpose of the CPTs overall is to measure attention, vigilance, and inhibition. To measure these functions, there are two types of stimuli involved in the test. These are referred as target and non-target stimuli. Participants are assigned a target at the beginning of the test. This target may be a specific symbol or any stimuli and the scoring is performed by using omission and commission errors. The scoring would be done through omission and commission errors. Comission errors refer to pressing the button for a symbol other than the target and omission errors occurs when the target is missed (Sparrow, 2010). Through these values, certain functions such as attention, vigilance, inhibition and working memory could be measured (Harpin et al., 2016).

A recent systematic review and meta-analysis reviewed 74 studies using various neuropsychological measures, with most focusing on CPTs to evaluate attention, impulsivity, and response time variability. As seen in most CPT measurements, the CPTs analyzed commonly tracked variables such as omission errors (indicating inattention), commission errors (impulsivity), and reaction time variability. The SN of these tests varied widely, from 22 to 100%, with SP also ranging between 22 and 100%. The AUC values ranged from 0.59 to 0.93, showing the mixed performance of these tools (Peterson et al., 2024).

Just as any other tool, CPTs come with some limitations. One of these limitations particularly embodies validity and reliability issues. These tools, although being practical, may lack reliability due to technical factors, such as issues with the software or hardware used during administration (Roebuck-Spencer et al., 2017). Some CPTs can be financially burdening as they often require specialized equipment, and training. Additionally, CPTs measure limited abilities or only a sample of behavior under a controlled environment. And the CPT results of an individual may still not be reproducible in the same contexts due to several other uncontrollable factors such as motivation, attention or the type of existing disorder. Hence, the CPT results may not necessarily reflect the ADHD symptoms or may be influenced by other factors which are not directly related to the disorder. These factors could also introduce false positives that are either reporter or context-dependent which also indicates the importance of using appropriate normative data to ensure reliable interpretation (Rabin et al., 2014). Additionally, the application of multiple tasks may be particularly challenging for individuals with ADHD, who may have difficulty maintaining focus and remaining still for long periods of time (Breitling-Ziegler et al., 2020). Additionally, CPT’s ability to identify a wide range of deficits may be limited, as most of them are sensitive to identify specific types of functions such as attention and inhibition (Onandia-Hinchado et al., 2021). Collectively, these limitations highlight the importance of the clinical utility of these tools, as illustrated in Table 4 through SN and SN values of some of the most employed CPTs in ADHD:

**Table 4:** Common CPTs used in ADHD assessment: key characteristics and diagnostic accuracy.

CPT	Domain	Duration and age range	Parameters	SN & SP (95% Cl)	SN SP source
Test of Variables of Attention (TOVA) (Greenberg and Waldmant, 1993)	Attention and inhibitory control	15-20 min4 to 80 years	Omission and commission errors, RT, RTV, D prime (response sensitivity), attention comparison score, post-commission response time, anticipatory responses, multiple responses	SN: 0.77 [0.62, 0.87] SP: 0.68 [0.49, 0.83]	Pooled results (Arrondo et al., 2024)
The Quantitative Behavioral Test (QbTest; http://www.qbtech.com)	Attention, impulsivity and hyperactivity	20 min 6 to 60 years	QbInattention: omission Errors, RT, and RTV QbActivity: time active, distance, area, and micro events QbImpulsivity: commission errors, normalized commission errors, and anticipatory responses ADHD total score	SN 0.48 [0.35; 0.61] SP 0.83 [0.60; 0.94] SP: 0.65 [0.48; 0.78] SN: 0.65 [0.52; 0.75] SN: 0.49 [0.33; 0.65] SP: 0.76 [0.63; 0.86] SN 0.78 [0.69; 0.85] SP 0.70 [0.57; 0.81]	Pooled results (Bellato et al., 2024)
MOXO (https://moxo.ai/)	Attention, impulsivity, hyperactivity, and timing	15 min 6 to 65 years	ADHD scale omission errors, commission errors	SN 0.82 [0.64, 0.92] SP 0.87 [0.83, 0.90] AUC 0.57-0.64	Pooled results (Arrondo et al., 2024)
Conners CPT-3 (Conners et al., 2018)	Attention, vigilance, sustained impulsivity	14 min 8+ years	Omission errors, commission errors, variability, detectability, perseveration, response style, HRT, HRT SD, HRT block change, HRT ISI change	SN: between 0.19–0.41 SP ≥ 0.90 AUC: 0.62–0.69	Limited utility in detecting invalid performance (Callan et al., 2024)
IVA-2 (Sandford and Sandford, 2016)	Visual attention, auditory attention and impulse control	20 min 6 to 99 years	Response control Attention Attribute Symptomatic	SN: 0.75 [0.51, 0.90] SP: 0.68 [0.56, 0.78]	Pooled results (Arrondo et al., 2024)

AUC, Area Under the Curve; CI, Confidence Interval; SN, Sensitivity; SP, Specificity; HRT, Hit Reaction; Time; RT, Response Time; RTI, Reaction Time; RTV, Response Time Variability; RVP, Rapid Visual Information Processing; SD, Standard Deviation; SWM, Spatial WM; IED, Intra-Extra Dimension Set Shift; IVA-2, Integrated Visual and Auditory Continuous Performance Test, Second Edition; VRM, Verbal Recognition Memory.

MOXO, TOVA, QbTest, Conners CPT-3 and IVA-2 show varying psychometric utility (see Table 4) based on meta-analyses and experimental studies. In particular, the MOXO and TOVA show high SE (82 and 77%, respectively) and SP (87 and 77%), indicating that they are reliable in assessing ADHD-related symptoms such as attention, impulsivity and hyperactivity. But these findings are based on pooled data, which may not always reflect the diversity of real-world clinical settings. The QbTest provides significant utility in some subscales, however SN and SP vary across its subscales. Conners CPT-3, despite its high SP (≥90%), has a low SN (19–41%), suggesting that it may be more useful in ruling out ADHD diagnosis. The IVA-2 provides valuable insight into both visual and auditory attention, although its moderate SN and SP (75 and 68%) may limit its role in screening.

CPTs could be recommended to be used as a complementary tool, such as for monitoring treatment progress or as part of the diagnostic process alongside rating scales or previous medical and academic reports, can strengthen the evaluation process. For instance, QbTest have shown that attention measure of the QbTest is linked to overall impairment profile in ADHD and demonstrates sensitivity to the effects of ADHD medication (Emser et al., 2018). Finally, the applicability of CPTs for individuals with mental health challenges or disabilities requires further investigation to determine its effectiveness across diverse populations (Lancaster et al., 2009).

CPTs can be a useful tool for evaluating EF in ADHD (Park et al., 2019) when interpreted with caution, and the clinical utility of these tools is carefully considered. Besides, they may fail to represent the full EF profile since these tests are conducted in structured environment (Krieger and Amador-Campos, 2018). It is fundamental to emphasize that EF impairments are not exclusive to ADHD and may also occur in other disorders. While the CPT is effective in identifying cognitive differences between healthy individuals and those with ADHD, it may not be as reliable for distinguishing ADHD from other psychopathologies. While CPTs are useful for identifying cognitive deficits in ADHD, their variability suggests that they should be combined with other diagnostic methods, as they alone should not be used as a diagnostic tool (Peterson et al., 2024; Arrondo et al., 2024). CPTs could be recommended to be used as a complementary tool, such as for monitoring treatment progress or as part of the diagnostic process alongside rating scales or previous medical and academic reports, can strengthen the evaluation process. For instance, QbTest have shown that attention measure of the QbTest is linked to overall impairment profile in ADHD and demonstrates sensitivity to the effects of ADHD medication (Emser et al., 2018). Finally, the applicability of CPTs for individuals with mental health challenges or disabilities requires further investigation to determine its effectiveness across diverse populations (Lancaster et al., 2009).

In summary, both neuropsychological tests and CPTs are widely used and acknowledged in the ADHD assessment. Nevertheeless, there are important differences in ADHD presentation between children, adolescents and adults in terms of symptom expression, which requires different diagnostic measure considerations. In children, as hyperactivity is more overt and observable, with objective CPT measures like movement patterns and reaction times may be very effective for assessing hyperactivity. In contrast, hyperactivity presentation in adults implies more internal restlessness rather than a noticeable behavior. Hence, for adult assessment, rating scales may be more ideal and relevant (Emser et al., 2018). More so, ADHD encompasses a range of impairments with different intensities due to its heterogeneous nature. Some individuals may exhibit severe deficits in EF, while others show difficulties in non-executive processes like memory, temporal processing, and motivation regulation (Faraone et al., 2015). These findings not only contradict the notion that executive dysfunction is a core feature, but also may attention regarding the accuracy of tests focusing solely on cognitive measures.

## 5 Potential issues in ADHD diagnosis

### 5.1 The DSM

One of the most discussed problems with the DSM is the age of onset of ADHD. It has been widely discussed that ADHD is an early age onset disorder, but the concept of “late-onset ADHD” is also debated in the literature (Caye et al., 2017; Cooper et al., 2018). The previous versions of DSM involved statements more likely to apply to children and adolescents. However, the most recent version of DSM (DSM-5) has expanded these statements and given examples to the statements involving all age groups. For instance, in the DSM-IV, the statement “often loses things necessary for task activities” applied to toys, school- related activities or items. In DSM-5, these examples were expanded to items which also apply to adults (e.g., wallets, paperwork, keys). Conversely, a significant number of adults with ADHD do not receive a diagnosis until later in life (Johnson et al., 2020) which could be due to the age criteria in the previous versions. Nevertheless, since the DSM-5 transition, ADHD diagnoses in young adults have risen by 27% (Matte et al., 2015). The rise in the number of ADHD diagnoses may be positive indicator for improved access for clinical support. Regardless, it also raises concerns about potential “over-diagnosis.” The findings of a scoping review further reinforced this idea, showing increase in diagnoses and pharmacological treatment (Kazda et al., 2021), With the changes in DSM-5 and changing trends in ADHD, it is difficult to determine whether ADHD is over-diagnosed, overtreated or misdiagnosed. Such a condition may have serious consequences, such as potential substance abuse as a result of pharmacological treatment, economic burden on the society as well as the need for new regulations and an updated diagnostic system (Manos et al., 2017).

Considering the changes in the age-onset criteria, diagnosing ADHD in adulthood could be challenging. This is particularly due to the self-report assessment of the childhood symptoms. Adults could struggle recalling the onset of the symptoms, as this part of the assessment heavily relies on retrospective memory (Pallanti and Salerno, 2020).

Moreover, Posner et al. (2020) reported that ADHD is becoming recognized as a dimensional condition, rather than being categorically separated as mild, moderate or severe from non-ADHD individuals, as the DSM-5 states. They further discuss the absence of dimensional models and the questionable practicality of the categorical framework. Additionally, they emphasize that the DSM-5 overlooks the changes in the ADHD presentation across developmental stages. Similarly, inattention symptoms persist into adulthood while visible signs of ADHD often tend to lessen although it is unclear whether this is due to the diagnostic criteria or absolute remission of the symptoms (Faraone et al., 2021).

A recent review by Sadek (2023) emphasized another important limitation of the DSM. The criteria for ADHD in the DSM-5 do not specify that the symptoms are not “substance-induced or are attributable to the physiological effects of another medical condition,” as is written in the criteria for Major Depressive Disorder (MDD). Conditions such as sleep-disordered breathing, thyroid dysfunction, diabetes and typical absence seizures could resemble some ADHD symptoms, potentially resulting in serious consequences. Therefore, consulting a medical practitioner and conducting blood tests to rule out any, the importance of consulting a medical practitioner and running blood tests to rule out any underlying medical conditions is essential.

### 5.2 Impact of comorbidities

#### 5.2.1 Comorbidity and ADHD

Adult ADHD is highly susceptible to misdiagnosis or a secondary diagnosis due to comorbidity or resemblance to other conditions. ADHD symptoms in early life may be masked due to the similarity to anxiety, mood or substance use disorders (Anbarasan et al., 2020). Results from a longitudinal study indicated that ADHD is a challenging disorder with a high rate of comorbidity (Reale et al., 2017). Most comorbid cases in ADHD include conduct disorder (CD), oppositional defiant disorder (ODD), learning disorders (LD), anxiety, mood disorders (MD), autism spectrum disorders (ASD), and tic disorders (TD) (Elwin et al., 2020).

There is a notable prevalence of co-occurring ADHD symptoms in children diagnosed with Autism Spectrum Disorders (ASD). This comorbidity is linked to an elevated risk of amplified psychosocial difficulties. Both ASD and ADHD present commonalities in their clinical profiles, including communication difficulties, restricted behaviors, and attention problems (Harkins et al., 2022). EF deficits are recognized as transdiagnostic factors contributing to both ASD and ADHD. Inhibition emerges as a key area of difficulty, potentially linked more strongly to ADHD symptoms. Similarly, working memory deficits also may be present as one of the challenges in ASD (Luderer et al., 2021). Moreover, both disorders exhibit a higher prevalence in boys compared to girls (Coulacoglou and Saklofske, 2017).

Diagnosing ADHD and ASD together poses unique difficulties due to overlapping symptoms and limitations in standard diagnostic tools. While ADHD rating scales and structured interviews are effective for ADHD, they may not reliably distinguish ADHD symptoms in individuals with ASD (Bölte et al., 2018). To prevent these overlaps, several tools like the Social Communication Questionnaire (SCQ) and the Autism Mental Status Examination (AMSE) have shown better accuracy (SN: 0.83, SP: 0.90) in differentiating ADHD from ASD and their comorbid presentations. On the other hand, Kiddie Schedule for Affective Disorders and Schizophrenia for School Aged Children Lifetime Version (KSADS-PL) and behavioral observations could aid in the diagnostic process (Antshel and Russo, 2019). However, clinical judgment remains critical in interpreting these assessments, as the distinctions between ADHD and ASD symptoms are often blurred.

Conduct Disorder (CD), has been identified as one of the most prevalent comorbid conditions, with ADHD in 10–20% of cases (Sadek, 2014). The family history of ADHD and CD comorbidity increases the risk of recurrence. Similarly, at the cognitive and neuronal level, both CD and ADHD share common challenges such as inability to regulate and process emotions and poor decision-making which supports the concurrent occurrence. Additionally, ADHD and CD have similarities in negative personality traits as low self-regulation skills in individuals with ADHD lead to negative emotions such as irritability and anxiety and these negative traits are also very common in CD. Finally, CD is more likely to be reported than ADHD due to externalized symptoms (Thapar and van Goozen, 2018). However, that the risk of CD comorbidity depends on the severity of ADHD symptoms.

Alternatively, oppositional defiant disorder (ODD) is an externalized disorder is frequently reported in ADHD. The approximate prevalence of ODD comorbidity in ADHD is 60% and for CD the prevalence is around 20%. The co-occurrence of ODD and CD in ADHD is highly dependent on age and sex (Hudec and Mikami, 2017). Both CD and ODD comorbidity in childhood ADHD is very frequently reported and more common among boys (Azeredo et al., 2018). ADHD, ODD, and CD symptoms develop together from late childhood to adolescence and that there is a high likelihood of experiencing further escalation in ADHD, ODD, and CD symptoms with time (Atherton et al., 2020). Although early diagnosis of ADHD, ODD, and CD in the preschool period is difficult due to the symptom similarities of these disorders. However early diagnosis can positively change the developmental course of these disorders.

Learning disorders (LD) are also known to challenge individuals with ADHD throughout their educational and professional life. LD involves disorders such as dyslexia (reading difficulties), dyscalculia (difficulties in mathematical skills) and dysgraphia (writing difficulties). Children with LD often feel helpless and act out and are often unable to regulate their emotions. The rate of co-occurrence of LD with ADHD is very high (U.S. Department of Health and Human Services, 2011).

ADHD is also very frequently accompanied by mood disorders such as bipolar disorder, depression, and dysthymia as well as anxiety disorders. The prevalence of depression and ADHD was reported to be at an average of 7.8% (Katzman et al., 2017). Comorbid depression occurs in ADHD in a regardless of gender and has a more negative impact on quality of life and cognitive functioning than in individuals with ADHD alone (Roy et al., 2017).

The prevalence of comorbid anxiety is significantly high in ADHD. A study reported that 55% of college students have one and 31.8% have two more comorbid disorders predominantly being anxiety and mood disorders (Anastopoulos et al., 2018). Although genetics is associated with comorbidities, the influence of environmental factors is quite serious. Psychosocial challenges experienced by individuals with ADHD at the school age may lead to the emergence of mood disorders or anxiety comorbidities. The problems caused by ADHD symptoms usually starting at school age, such as inability to concentrate in class, receiving low grades, extreme physical restlessness, breaking rules due to unrestrained impulsivity and being bullied by peers, lowers school persistence and academic success and may cause low self-esteem, depression and anxiety (Maltezos et al., 2020; Lung et al., 2019). Conversely, self-esteem is significantly lower in individuals with ADHD than in typically developing individuals. The fact that the symptoms interfere with many areas of their lives lead these individuals to be more prone to lack of self-confidence, sensitivity to rejection, social withdrawal which may aggregate the likelihood and vulnerability to developing mood and anxiety disorders (Beaton et al., 2022).

Obsessive compulsive disorder (OCD) and ADHD share many similarities in terms of neuropsychological aspects. Underactive EF is observed in both disorders (Rothenberger et al., 2018). As inhibition and impulsivity problems in ADHD have many negative consequences, these key problems also account for repetitive behaviors and intrusive thoughts in OCD. It is also stated that the comorbidity of ADHD with OCD is due to genetic transmission (Brem et al., 2014).

In case of Tic Disorders (TD), the visual dominance and prominence of TD leads to the disregard or oversight of existing ADHD (Rothenberger et al., 2018). The co-occurrence of ADHD and tic disorders is one of the most challenging comorbidities. This is due to the aggravation of tics causing difficulties in social life (Bélanger et al., 2018). Often, this situation may also contribute to the development of mood and anxiety disorders (Huisman-van et al., 2019).

Moreover, individuals with ADHD have a much higher likelihood of engaging in risky behavior than normal individuals. Between 25 to 40% of ADHD cases are involved in substance misuse and besides endangering the life of the individual, substance dependence has been associated with car accidents, engagement with dangerous behavior and suicidal ideation or attempt (Luderer et al., 2021; Wilens et al., 2018). One of the major contributing factors to this is their inability to make rational judgments, which can be explained by impaired EF (Vassileva et al., 2019). Impulsivity, lack of self- inhibition as well as the impaired brain reward system show how easily they may lapse into negative habits and quickly adapt to them (Ivanov et al., 2022; Blevins et al., 2020). Therefore, smoking, alcohol, substance abuse and even addiction are very common in individuals with ADHD. In addition, addiction is frequently reported in individuals with ADHD who use psychostimulants for the purpose of treatment due to taking more than the prescribed dose of the drug (Flores-García et al., 2020).

##### 5.2.1.1 Does comorbidity impact diagnostic accuracy?

It is crucial to acknowledge that ADHD symptoms could be coincident with those indicative of other disorders, due to the fact that numerous disorders exhibit symptoms similar to those of ADHD. And in most cases, this leads to misdiagnosis. It is therefore very important to determine how long the symptoms have been persistent. It is certainly very important to detect how long the symptoms have been present (Maltezos et al., 2020) which is usually recognized during rapport building. To ensure the accuracy of the diagnosis, the risk of comorbidity should be analyzed in line with the structured interview. Additionally, for disorders that are complex to distinguish, such as the comorbidity of anxiety and ADHD, it is useful to utilize rating scales that are reliable to provide a more valid diagnosis (Grogan et al., 2018).

In terms of misdiagnosis, anxiety, depression are usually the most common reported conditions. This is because restlessness, attention problems and forgetfulness are some of the many symptoms seen in both anxiety and depression in ADHD. Additionally, ADHD may sometimes be mistaken for bipolar disorder. However, there are distinctive clinical symptoms. For example, both disorders are characterized by mobility, distractibility, insomnia, irritability, anger outbursts, mood swings and social assertiveness. However, the distinctive features in bipolar disorder are excessive cheerfulness, self-confidence, increased sex drive, unwillingness to sleep, racing thoughts, pleasure to engage in risky activities and suicidal thoughts or attempts. However, these symptoms are not seen in ADHD. Conversely, social interaction, affective problems and speech delay are the features that are indicated in both ASD and ADHD. However, social problems, forming friendships, and cognitive development are much slower in ASD than in ADHD. In order to distinguish these, the level of dysfunction must be assessed (Bölte et al., 2018). It must not be ignored that comorbidity is a very sensitive concept in diagnosis. Although some disorders are interpreted as co-existing, misdiagnosis or under-diagnosis should not be overlooked (Ginsberg et al., 2014).

### 5.3 Gender bias in diagnosis

Diagnostic criteria for ADHD have traditionally been developed using predominantly male child samples. ADHD in women has been often un-recognized due to gender bias in the diagnostic procedure. Females are less likely to be referred for an assessment and they often receive misdiagnosis (Danielson et al., 2018). Notably, that women are more likely to report inattention symptoms, and less hyperactivity or impulsivity compared to boys (Antoniou et al., 2021). Males overall have a higher diagnosis ratio than females which could be due to the under-diagnosis of ADHD in females (Sadek, 2014).

Even though ADHD is believed to be more common in males, it is emerging to be highly recognized in females (Faheem et al., 2022). ADHD is usually diagnosed at an early age, with early signs in toddlers being noticeable between 2 and 3 years of age through irregular sleep patterns (Posner et al., 2020). However, at early ages, the hyperactivity symptoms tend to be more pronounced, and it is a highly predominant symptom in boys. In girls, on the other hand, internalized symptoms; more inwardness, shyness, and inattention are observed (Attoe and Climie, 2023).

Because of the expression of the symptoms, it is more often observed that boys acquire ADHD diagnosis at a young age, much earlier than girls. Regarding older ages, especially in women, non- diagnosed ADHD is generally identified and treated as anxiety or depression (Hinshaw et al., 2022). Consequently, most women are diagnosed with ADHD at a later age (Fairman et al., 2020) and they are generally misdiagnosed, which may also be due to gender bias arising from the traditional nature of ADHD. In addition, as women with undiagnosed ADHD are more likely to experience heightened hormonal shifts that result in higher levels of irritability, emotional dysregulation, mood swings and poor concentration, they may be more likely to receive another diagnosis (Attoe and Climie, 2023). While neurocognitive profiles of adults with ADHD have been studied, gender differences in these cognitive patterns are often overlooked, despite biological and hormonal factors suggesting potential disparities between males and females. Thus, both men and women may be misdiagnosed due to differences in ADHD presentation and gender-related differences.

Recognizing the potential differences in the presentation of ADHD due to gender differences could help reduce the gender gap, however there is a need for improved diagnostic tools (Slobodin and Davidovitch, 2019). Among other tools, DIVA-5 is currently exploring new methods to improve the identification of ADHD symptom presentations in women. This includes investigating symptom differences between men and women and hormonal factors to improve the diagnostic accuracy for women.

### 5.4 Differential diagnosis

ADHD should not be diagnosed if symptoms are solely present during psychotic episodes or could be explained by another mental disorder. Individuals with autism or intellectual disabilities may be diagnosed with ADHD if their symptoms exceed the expected severity for their developmental level. Clinicians must distinguish ADHD from other mental disorders, especially those that share overlapping symptoms, by noting the persistence of inattentive or hyperactive–impulsive symptoms outside of discrete episodes of mood or anxiety issues. When both sets of diagnostic criteria are met, comorbidity should be considered (Hall et al., 2016).

As noted previously, some medical conditions like thyroiditis and diabetes may mimic ADHD symptoms (Sadek, 2023). Evidence-based clinical guidelines also suggest that the use of certain drugs or substances can be a leading factor in the appearance of ADHD-like symptoms and should also be addressed in the diagnostic process (May et al., 2023). Referral for a medical assessment (general examination, blood tests, brain imaging) and taking a medical history may also help to assess all of these factors.

In case of co-existing conditions, it is essential to undertake a thorough evaluation of both overlapping and distinguishing symptoms. To differentiate ADHD from other potential disorders, specific tests can be used in addition to interviews. For example, the Woodcock-Johnson IV (WJ IV), which measures achievement, cognitive abilities, and oral language, is an ideal scale for identifying potential learning disabilities (LD) (Schrank and McGrew, 2015). Similarly, for mood and anxiety disorders Child Depression Inventory (CDI) (Kovacs, 2015) and Revised Children’s Manifest Anxiety Scale, Second Edition (RCMAS-2) (Reynolds et al., 2008) could be utilized for detecting whether symptoms only mimic each other or there is comorbidity. Conversely, neuropsychological testing should also be considered for mapping out the impairments more accurately.

The functionality of the chosen tools for the assessment is also very important. First, in child and adolescent assessment, informant-based measures (parents and teachers) of EF, typically indicate greater delays in EF compared to performance-based measures. This discrepancy may arise because informant-based measures capture a child’s challenges in real-life contexts, such as school or home, where everyday demands refer to the difficulties in managing attention, impulsivity, and task completion. However, performance-based measures focus on isolated cognitive tasks that may not fully reflect the overall profile. However, using both measures in the diagnostic process to gain a comprehensive understanding of the child’s EF abilities (Sadozai et al., 2024).

Accurate diagnosis of ADHD requires a methodical approach, involving multiple steps that not only aid in identifying ADHD but also distinguish it from conditions with overlapping symptoms. Sibley (2021) outlines seven steps for accurately diagnosing adult ADHD. First step involves a structured diagnostic interview followed by the second step: self-report ratings and informant ratings. In this step, an utmost importance to informant ratings should be given as patients could over-report or under-report symptoms. To address this, the third step applies the “or rule,” which ensures that the symptom reported by the patient also needs to be endorsed by the informant to confirm its presence. The fourth step focuses on the areas of impairment and analyzing any existing evidence to support the impairment (e.g., academic and medical records, legal documents etc.) and the duration of these symptoms are assessed in the fifth step where the age-onset, the symptom timeline and potential stressors are further evaluated. In the sixth step, differential diagnoses are explored to rule out any alternative mental or medical conditions which may resemble ADHD symptoms. And finally, the last step allows for the establishment of a diagnosis. Following these diagnostic steps and addressing the differential diagnosis process, ADHD could be correctly identified, which would facilitate effective treatment and management.

## 6 Conclusion

In conclusion, diagnosing ADHD is a challenging and multifaceted process that requires gathering information from diverse sources, including standardized assessments and clinical observations, while considering the limitations of these tools as well as the diagnostic guidelines such as the DSM. Factors such as cognitive differences, age, gender, and comorbid conditions must also be accounted for to reduce potential biases in diagnosis.

A comprehensive, multimodal approach that combines subjective measures with objective tools is essential to capture the complexity of ADHD and minimize diagnostic errors. Such an approach not only improves the understanding of individual cases but also addresses concerns about over-diagnosis and under-diagnosis, which can arise from overlapping symptoms with other conditions. Utilizing assessments for EF screening, comorbidities, and functional impairments further enhances the accuracy and utility of the diagnostic process. The debate over the ecological validity and objective reliability of the tools used in ADHD assessment measures persists; while some advocate for performance-based assessments as more objective evaluations of EF, others highlight the value of informant-based measures in predicting real-world functional outcomes (Sadozai et al., 2024).

While this study has not focused on the gender-specific issues or neuroimaging, future research should discover the role and of other neuroimaging techniques such as fNIRS (Poliakova et al., 2023) and contribute to the area of gender-specific presentations of ADHD Additionally, thorough research into the psychometric properties of existing tools is needed to reduce financial burden caused by these tools and ensure reliable, evidence-based practices in ADHD diagnosis.

Finally, advancing our understanding of ADHD and improving diagnostic practices will not only enhance clinical outcomes but also support more targeted and effective interventions for individuals affected by this complex condition.

## Footnotes

1
www.divacenter.eu
